# Tuberculosis of the First Metatarsophalangeal Joint Treated With Metatarsal Head Resection and Anti-tuberculous Chemotherapy: A Case Report

**DOI:** 10.7759/cureus.85758

**Published:** 2025-06-11

**Authors:** Taro Kasai, Atsuhisa Yamada, Tetsuro Yasui

**Affiliations:** 1 Orthopedic Surgery, Faculty of Medicine, The University of Tokyo, Tokyo, JPN; 2 Orthopedic Surgery, Teikyo University Mizonokuchi Hospital, Kawasaki, JPN

**Keywords:** anti-tuberculous chemotherapy, first metatarsophalangeal joint, metatarsal head resection, skeletal tuberculosis, surgery, tuberculosis

## Abstract

Tuberculosis of the foot is an uncommon form of extrapulmonary tuberculosis, and tuberculosis of the first metatarsophalangeal (MTP) joint is extremely rare. We report the case of a healthy 31-year-old woman from the Philippines with a three-year history of swelling and pain in her left first MTP joint. Foot imaging revealed severe bone destruction and granulation tissue formation at the first MTP joint. Chest imaging showed no pulmonary involvement. *Mycobacterium tuberculosis* was identified from the granulation tissue at the first MTP joint, leading to a diagnosis of isolated tuberculosis of the first MTP joint. Surgical debridement, including resection of the first metatarsal head, was performed, followed by a six-month course of anti-tuberculous chemotherapy. Throughout the three-year postoperative follow-up period, the patient exhibited no symptoms or radiographic signs suggestive of infectious relapse. This case underscores the importance of considering skeletal tuberculosis in chronic monoarthritis of the first MTP joint, even in the absence of pulmonary symptoms. To our knowledge, previous reports of tuberculosis in the first MTP joint have been limited to short-term follow-up. This case demonstrates the effectiveness of combined metatarsal head resection and anti-tuberculous chemotherapy in achieving sustained infection control over the mid-term follow-up.

## Introduction

Tuberculosis remains a global health challenge, with extrapulmonary manifestations accounting for 10-15% of all tuberculosis cases [1]. Skeletal tuberculosis is rare, comprising less than 15% of extrapulmonary tuberculosis cases [2]. The clinical manifestations of skeletal tuberculosis, similar to those of other bacterial infections, include joint pain, swelling, and a limited range of motion. However, a characteristic feature of skeletal tuberculosis is its slow progression, with discharging sinuses potentially developing in chronic cases. Among skeletal sites, the foot is an uncommon location for only about 10% of all cases of skeletal tuberculosis [3,4], and involvement of the first metatarsophalangeal (MTP) joint is particularly rare [5-8].

In this report, we present a case of isolated tuberculosis of the first MTP joint in a young woman without pulmonary involvement, successfully treated with a combination of metatarsal head resection and anti-tuberculous chemotherapy. Surgical intervention was performed both to confirm a definitive diagnosis through biopsy and to treat advanced-stage disease characterized by prolonged symptoms, severe joint destruction, and a discharging sinus. The treatment course was completed successfully, with no recurrence observed during a three-year follow-up. To our knowledge, previous reports of first MTP joint tuberculosis have been limited to short-term follow-up [5-8]. This case is the first to describe a combined surgical and medical treatment with mid-term follow-up, providing valuable insights into the management of this rare condition, for which there is limited existing literature.

The patient was informed that data concerning the case would be submitted for publication and provided consent.

## Case presentation

**Figure 1:**
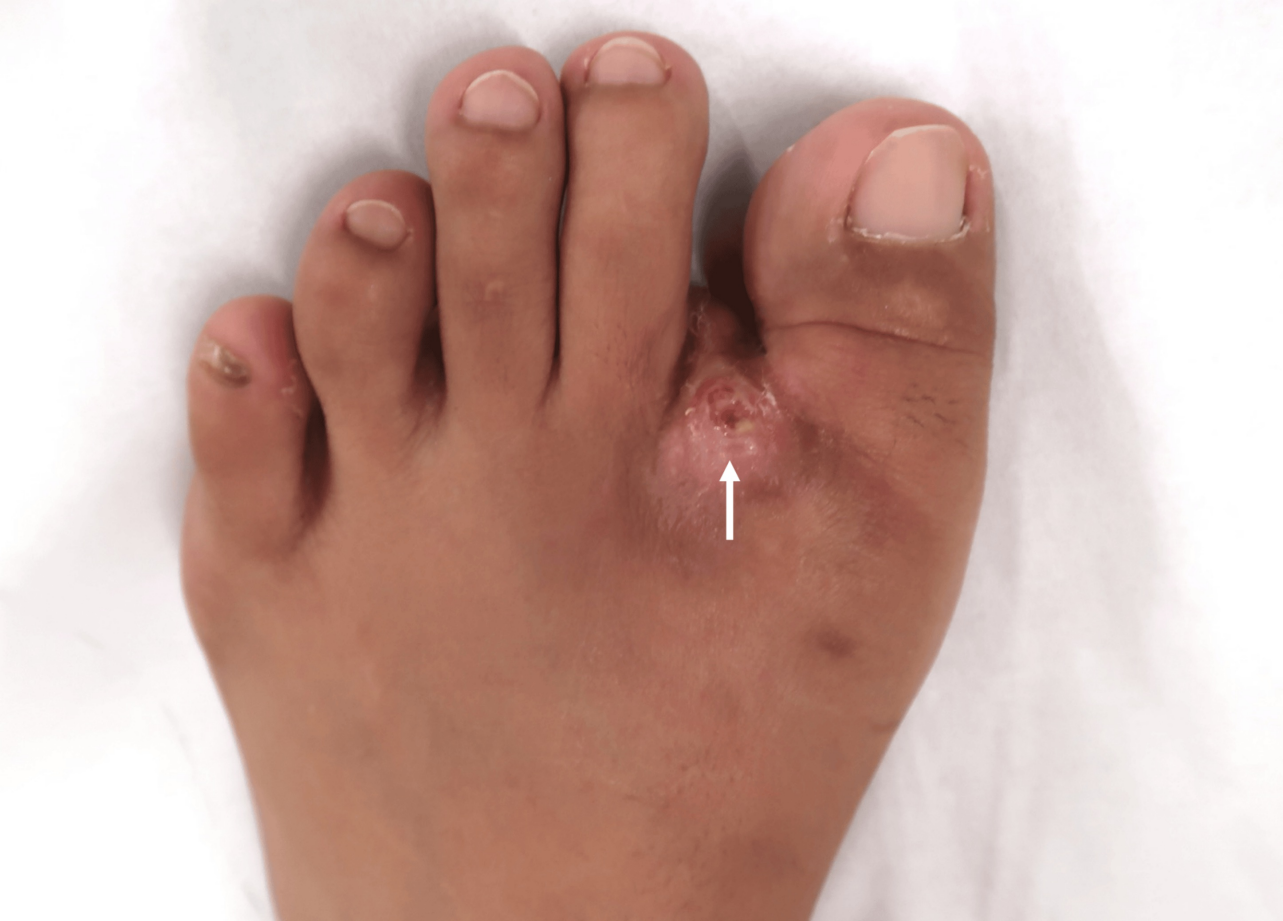
Preoperative photograph Preoperative photograph of the left foot demonstrating a discharging sinus on the dorsolateral aspect of the first MTP joint. MTP: metatarsophalangeal

A 31-year-old woman from the Philippines was referred to our institution due to a three-year history of swelling and mild pain in her left first MTP joint. She had been residing in the Philippines when the symptoms began. Approximately two years prior to referral to our institution, she immigrated to Japan, after which her symptoms gradually worsened, leading to the development of a discharging sinus on the dorsolateral aspect of the first MTP joint (Figure 1). She denied constitutional symptoms such as fever, weight loss, night sweats, or respiratory complaints. Her past medical history was unremarkable, and there was no history of trauma. Results of preoperative laboratory tests are summarized in Table 1.

**Table 1:** Results of preoperative laboratory tests

Parameter	Result	Reference range
White blood cell count	6,300/μL	3,500-9,000 /μL
C-reactive protein	0.07 mg/dL	<0.30 mg/dL
Erythrocyte sedimentation rate 1 hr	17 mm	0-20 mm (female)

**Figure 2:**
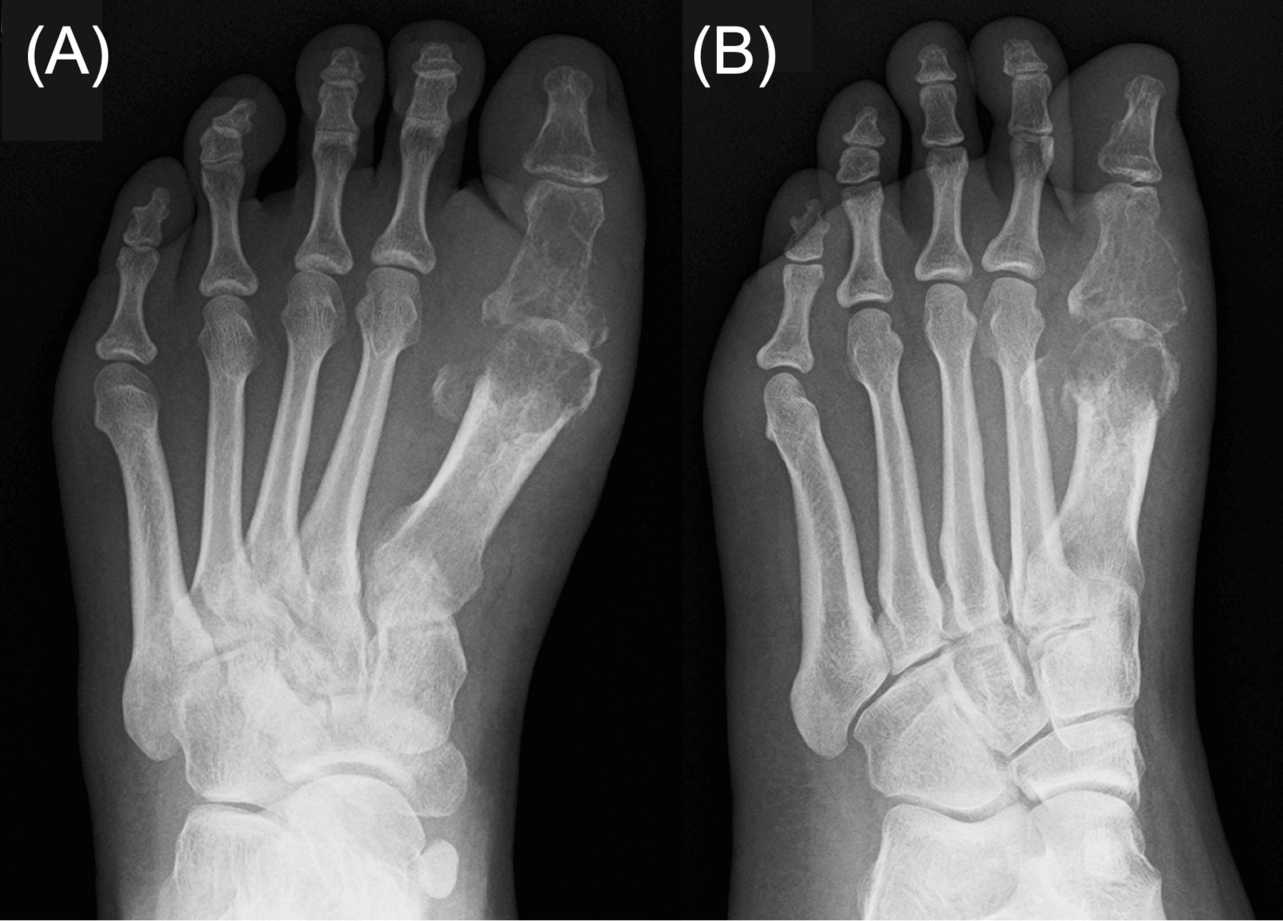
Preoperative plain radiographs Preoperative plain radiographs of the left foot in dorsoplantar (A) and oblique (B) views demonstrating severe destructive changes in the first metatarsal head and the proximal phalanx of the hallux.

**Figure 3:**
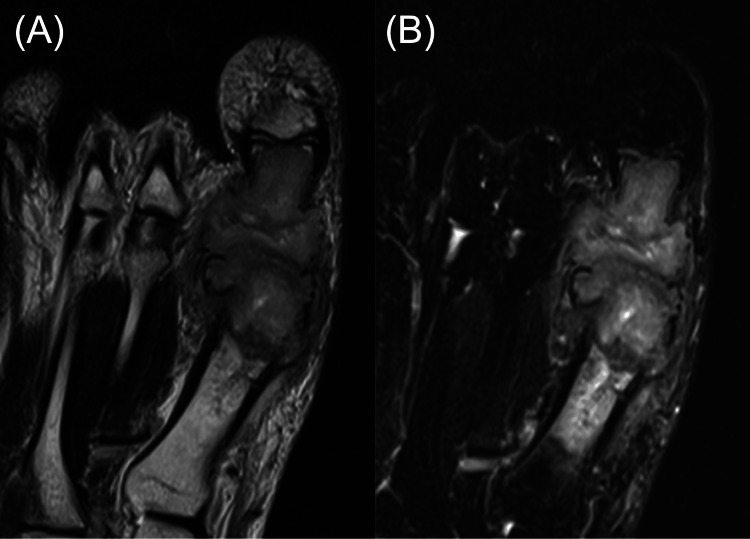
Preoperative MRI Preoperative non-contrast MRI of the left foot demonstrating granulation tissue proliferation at the site of joint destruction, as shown in T1-weighted image (A) and STIR image (B). MRI: magnetic resonance imaging, STIR: short tau inversion recovery

**Figure 4:**
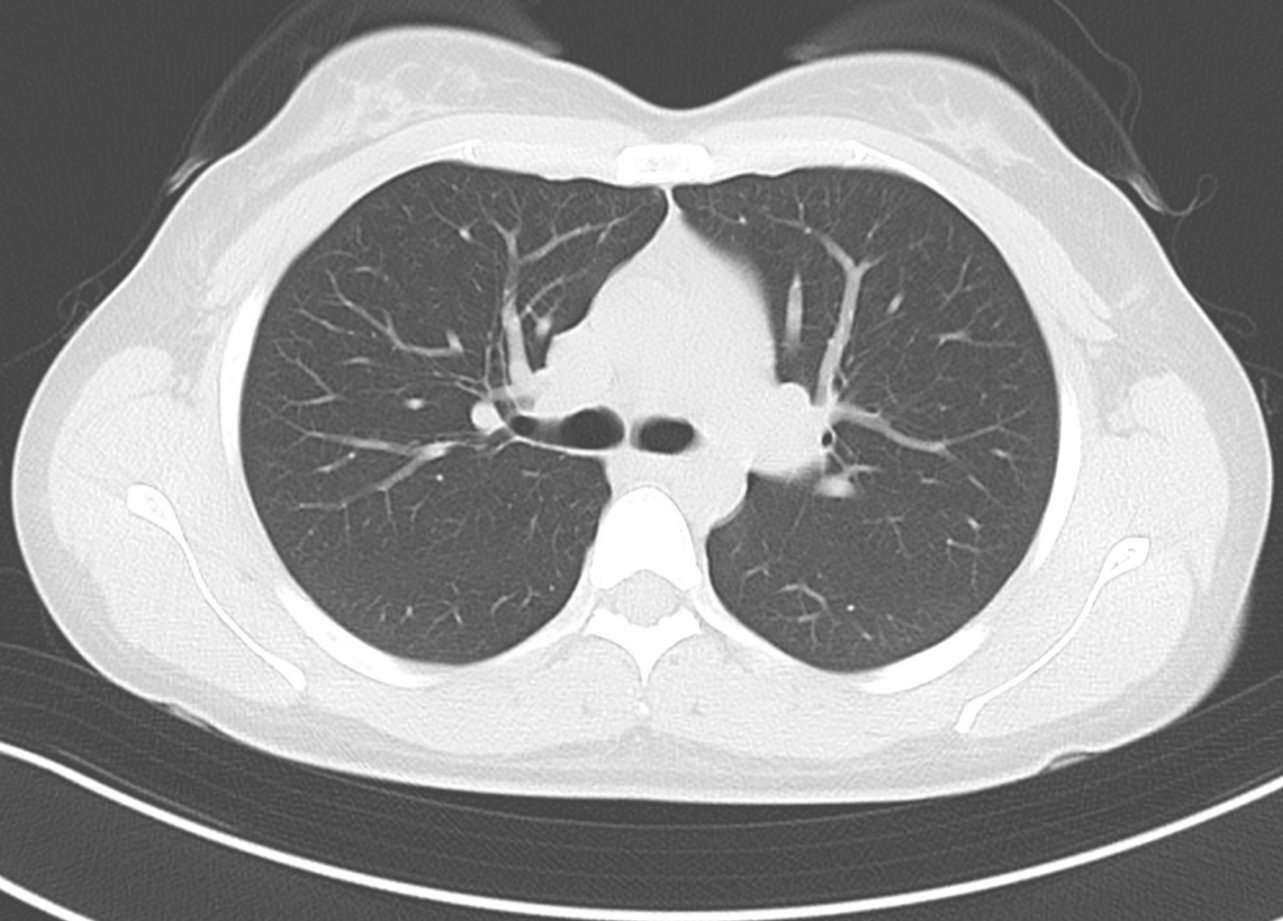
Preoperative chest CT Preoperative chest CT image demonstrating no findings suggestive of pulmonary tuberculosis. CT: computed tomography

Laboratory tests revealed a white blood cell count of 6,300/μL, a C-reactive protein level of 0.07 mg/dL, and a one-hour erythrocyte sedimentation rate of 17 mm, all within normal limits. Foot radiographs demonstrated destructive changes in the first metatarsal head and the proximal phalanx of the hallux (Figure 2), consistent with osteomyelitis. MRI demonstrated granulation tissue proliferation at the site of joint destruction (Figure 3). No pulmonary abnormalities were identified on chest computed tomography images (Figure 4).

**Figure 5:**
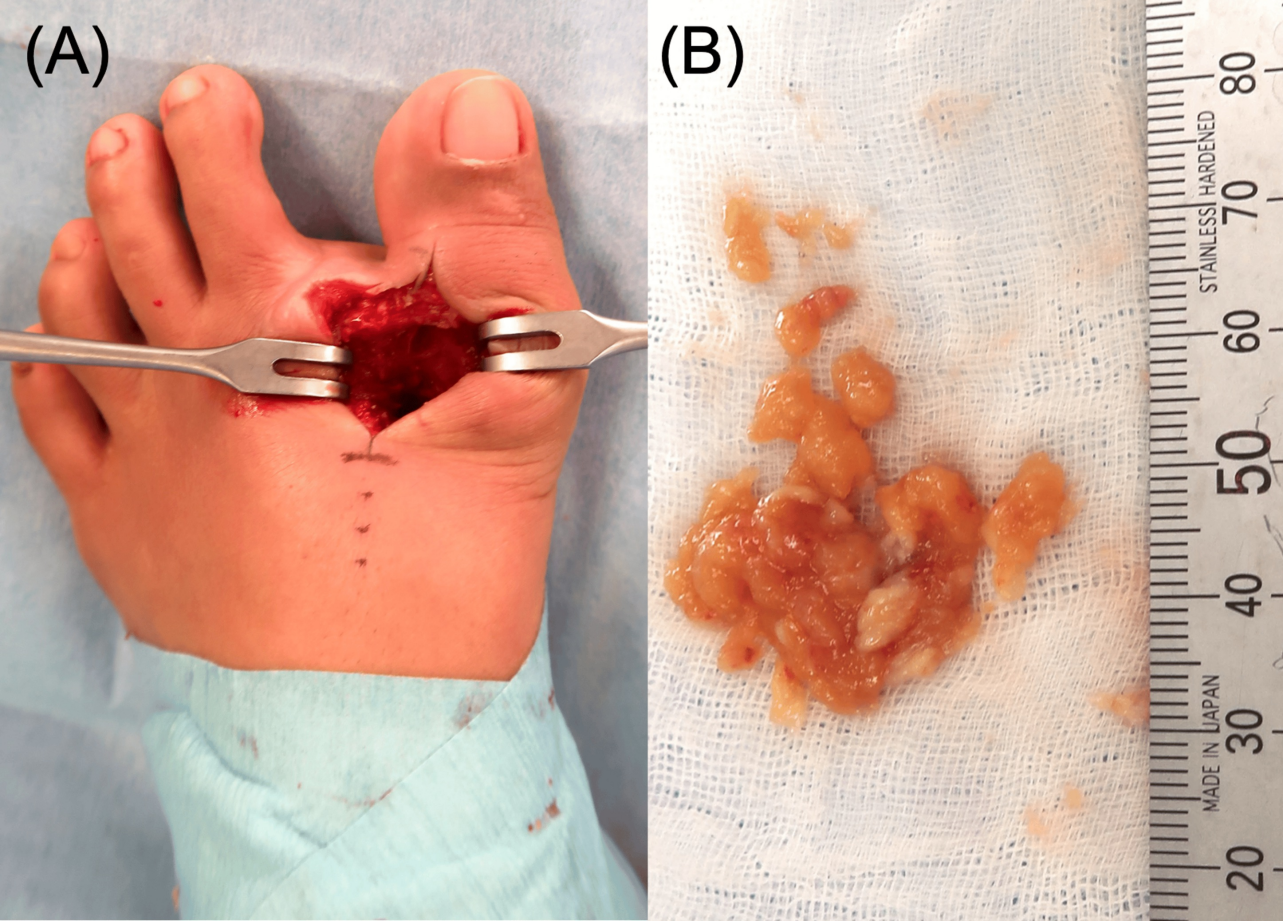
Intraoperative photograph Intraoperative photograph of the left foot demonstrating the first MTP joint after removal of the granulation tissue and destructed metatarsal head (A), and the collected granulation tissue (B). MTP: metatarsophalangeal

**Figure 6:**
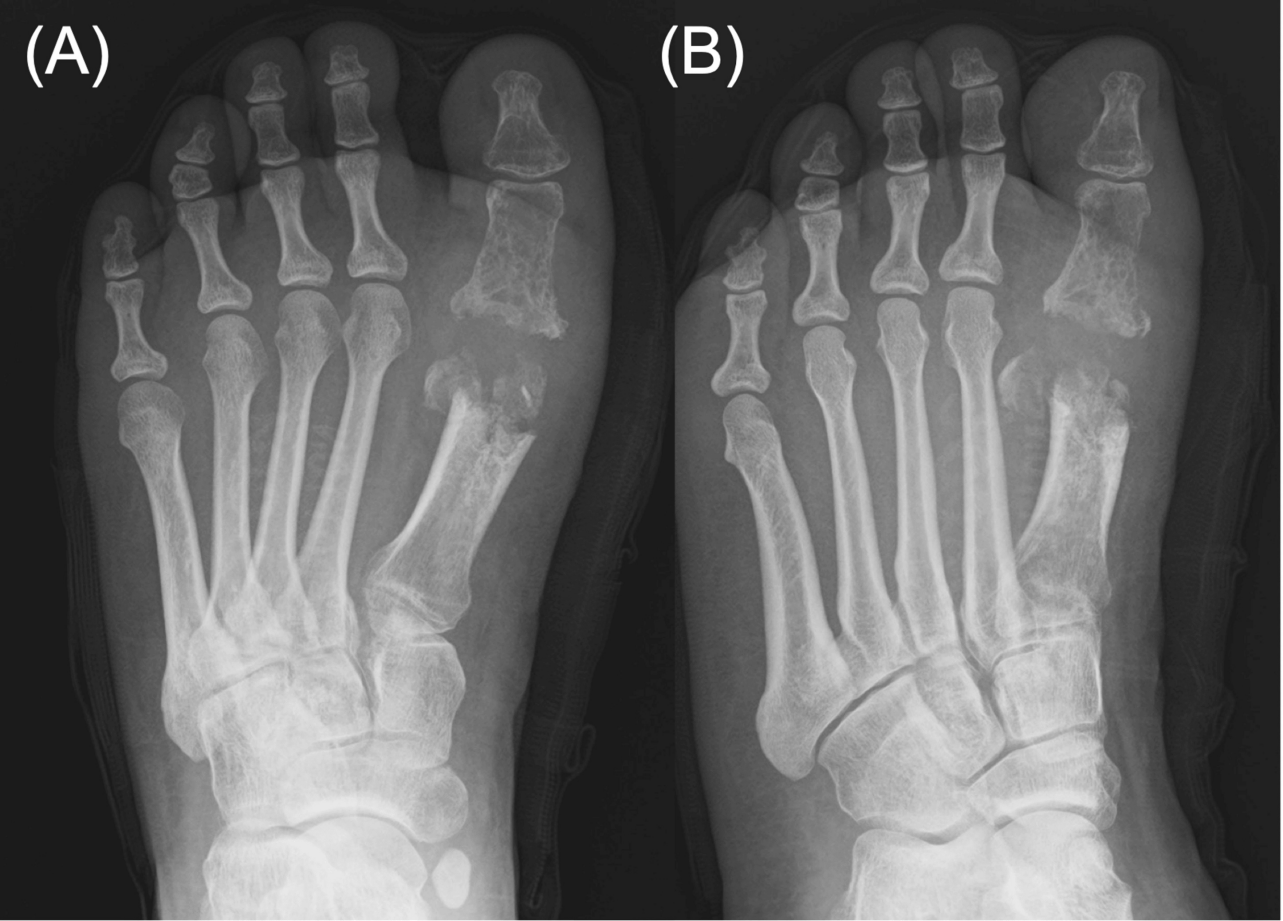
Immediately postoperative plain radiographs Immediately postoperative plain radiographs of the left foot in dorsoplantar (A) and oblique (B) views demonstrating the hallux with the metatarsal head surgically resected.

Surgery was performed for both definitive diagnosis and treatment of osteomyelitis. Under general anesthesia, a longitudinal incision approximately 4 cm in length was made on the dorsolateral aspect of the first MTP joint, excising the skin overlying the sinus. Subcutaneous dissection revealed proliferative granulation tissue extending to the MTP joint. The dorsal cortex of the proximal phalanx and the entire circumference of the first metatarsal head were found to be destroyed and replaced by granulation tissue. The granulation tissue was excised (Figure 5) and submitted for microbiological and pathological examinations, including cultures for common bacteria, *Neisseria gonorrhoeae*, *Cryptococcus*, fungi, and *Mycobacterium tuberculosis*. Polymerase chain reaction (PCR) testing for *Mycobacterium tuberculosis* was also performed. The residual first metatarsal head, which showed extensive destruction due to infection, was resected (Figure 6).

**Figure 7:**
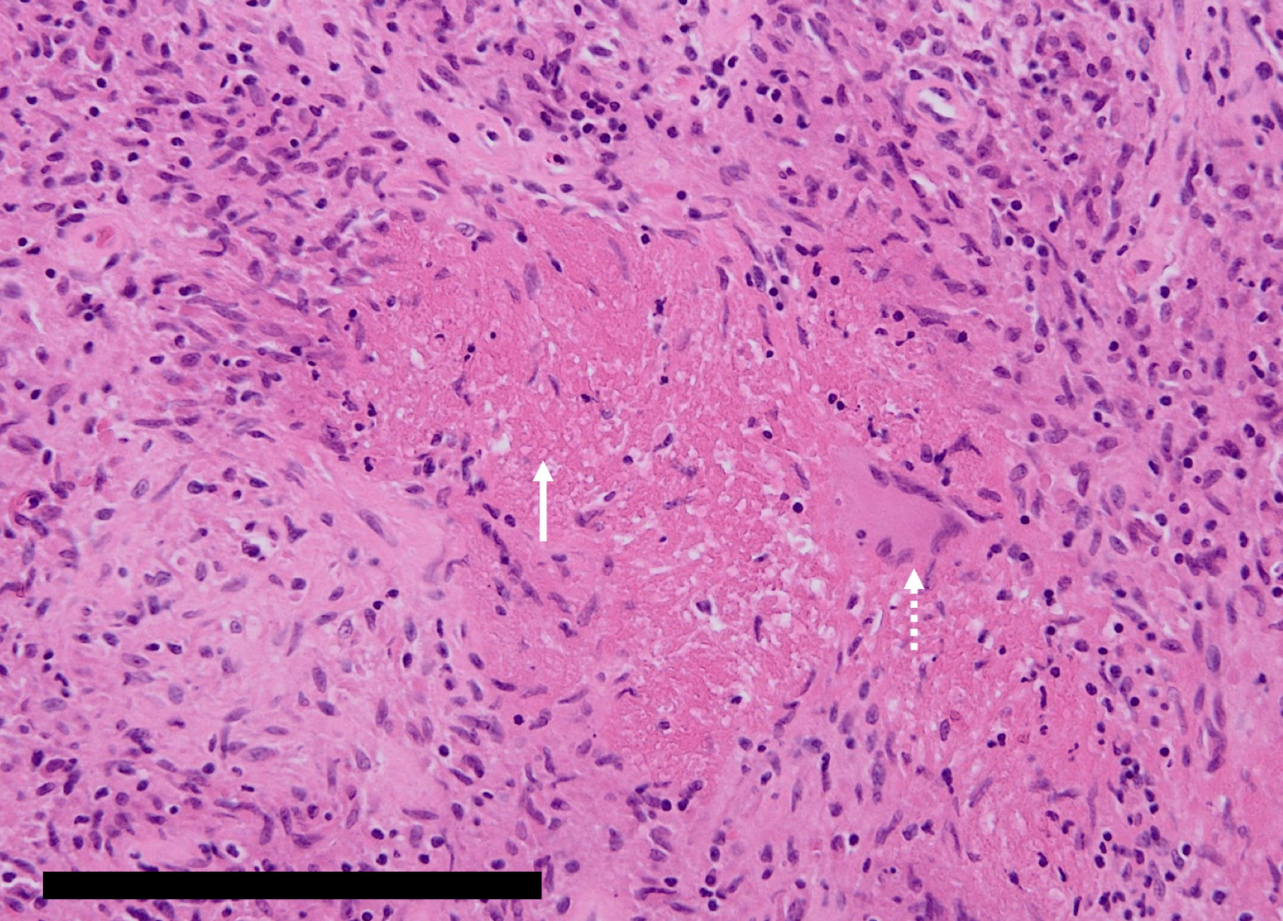
Histopathological examination Histopathological examination of the granulation tissue in hematoxylin and eosin staining demonstrating epithelioid cell granuloma with caseous necrosis (solid arrow) and Langhans-type giant cells (dashed arrow). Scale bar: 200 μm.

Histopathological examination of the granulation tissue demonstrated epithelioid cell granuloma with caseous necrosis and Langhans-type giant cells (Figure 7).

PCR and microbiological culture of the granulation tissue confirmed the presence of *Mycobacterium tuberculosis*. In contrast, sputum testing was negative for acid-fast bacilli. Therefore, the patient was diagnosed with isolated tuberculosis of the first MTP joint without pulmonary involvement. Postoperatively, the patient received a standard anti-tuberculous chemotherapy consisting of rifampicin, isoniazid, ethambutol, and pyrazinamide for 60 days, followed by a continuation phase with rifampicin and isoniazid for an additional 120 days, completing the treatment course. Postoperative care included the use of a forefoot offloading orthosis during ambulation for the first month. The surgical wound healed uneventfully within two weeks postoperatively.

**Figure 8:**
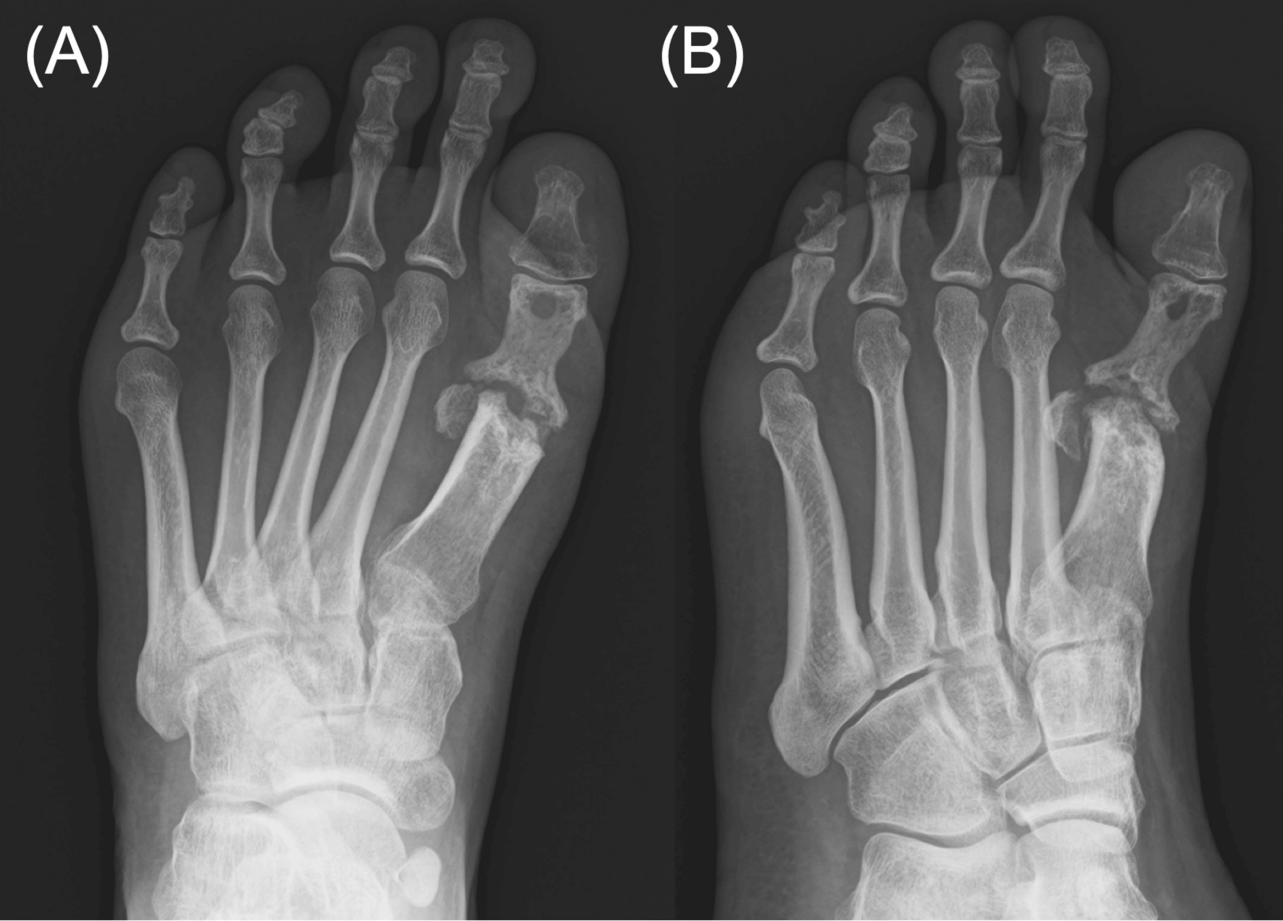
One-year postoperative plain radiographs One-year postoperative plain radiographs of the left foot in dorsoplantar (A) and lateral (B) views demonstrating bone sclerosis at the site of prior infection and shortening of the hallux.

At the one-year postoperative follow-up, there was no recurrence of pain or swelling. Foot radiographs demonstrated bone sclerosis at the site of prior infection without further bone destruction. The hallux was shortened as a result of metatarsal head resection (Figure 8).

**Figure 9:**
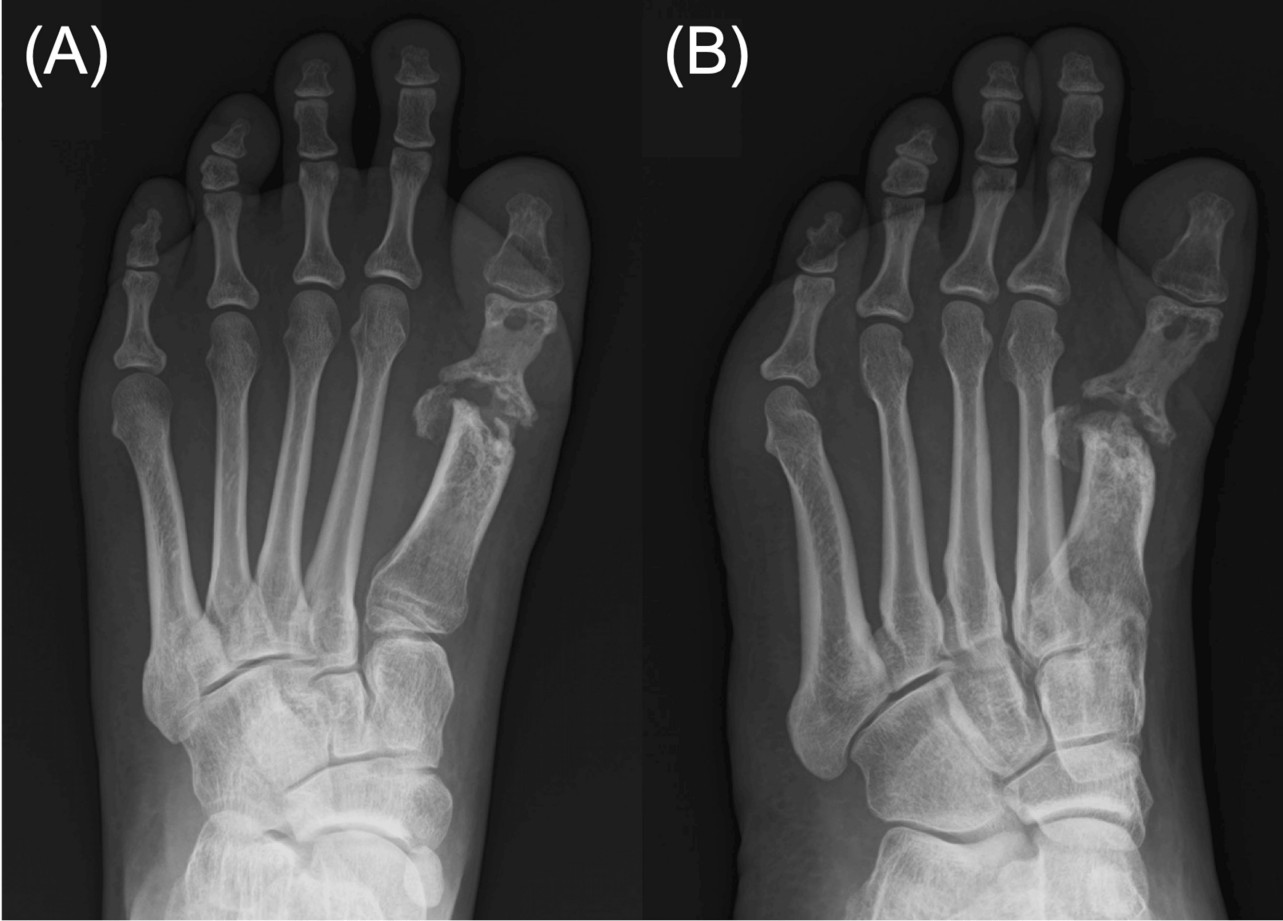
Three-year postoperative plain radiographs Three-year postoperative plain radiographs of the left foot in dorsoplantar (A) and lateral (B) views demonstrating persistent bone sclerosis without progression of bone destruction.

At the final postoperative follow-up at three years, the patient continued to show no recurrence of swelling or pain, and foot radiographs revealed no further progression of bone destruction (Figure 9).

**Figure 10:**
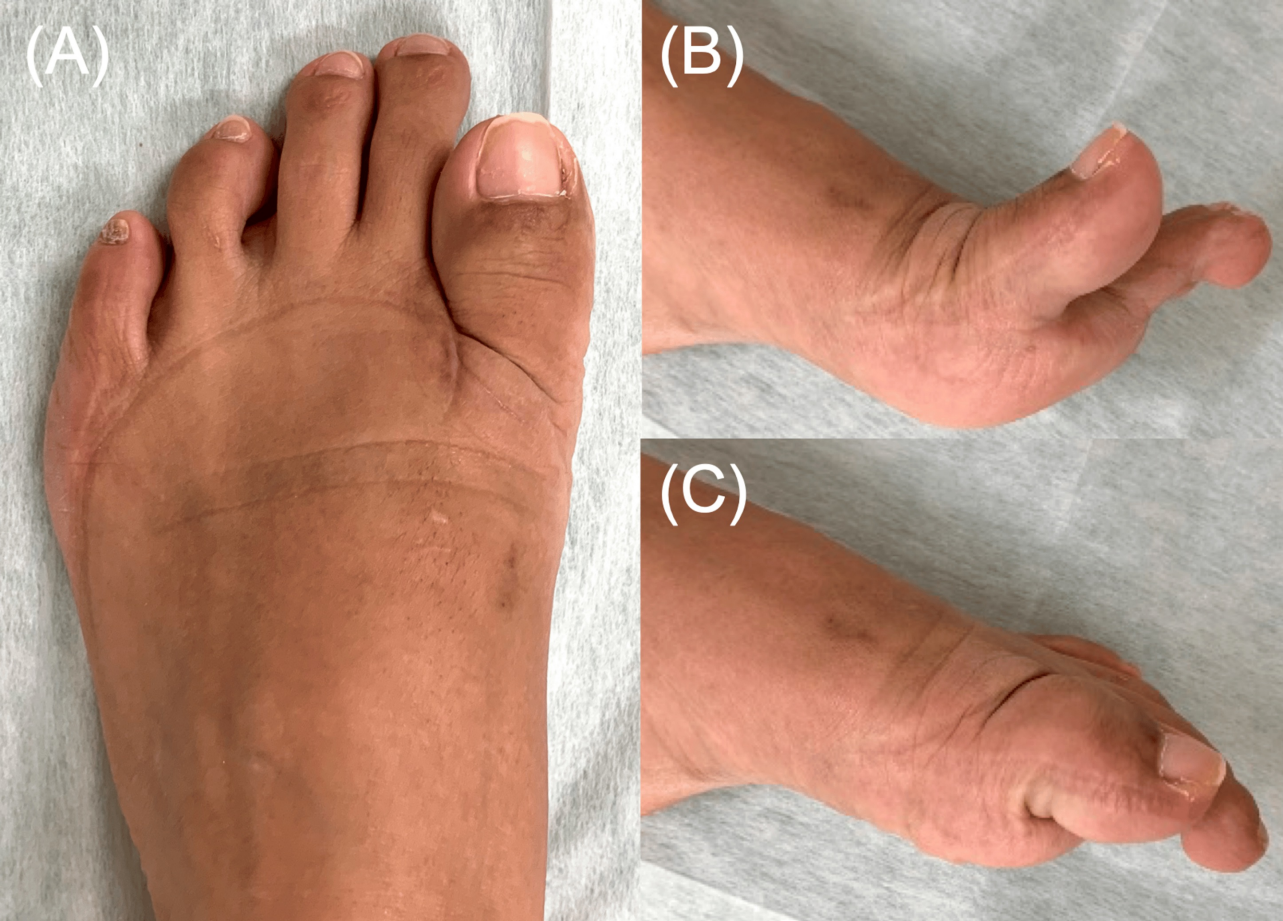
Three-year postoperative photographs Three-year postoperative photographs of the left foot. Dorsal view demonstrates shortening of the hallux (A). Lateral views demonstrate active extension (B) and flexion (C) of the first MTP joint. MTP: metatarsophalangeal

Active range of motion of the first MTP joint was 60 degrees in extension and 5 degrees in flexion (Figure 10). The patient reported no difficulty with daily activities or ambulation.

## Discussion

Skeletal tuberculosis often progresses slowly and insidiously, typically presenting with mild pain and low-grade fever [3]. Furthermore, fewer than one-third of patients with skeletal tuberculosis have concomitant active pulmonary tuberculosis [2]. Consequently, skeletal tuberculosis is often difficult to diagnose promptly. In particular, tuberculosis of the first MTP joint is exceedingly rare and may be misdiagnosed as pyogenic arthritis, gout, or rheumatoid arthritis, potentially resulting in delayed or inappropriate treatment [6]. In the present case, the infection was strictly localized to the first MTP joint, without any pulmonary findings or systemic signs, such as fever, making the diagnosis particularly challenging. However, the chronic course, the presence of a discharging sinus, progressive bone destruction, and the patient's origin from a tuberculosis-endemic region raised clinical suspicion for tuberculosis. The diagnosis was ultimately confirmed by the detection of *Mycobacterium tuberculosis* through PCR and culture testing of granulation tissue obtained from a surgical biopsy.

To the best of our knowledge, only four cases of tuberculosis involving the first MTP joint have been reported to date (Table 2) [5-8]. Patient ages ranged from eight to 80 years, and none were immunocompromised. Only one case exhibited concomitant pulmonary tuberculosis, while the other cases showed no evidence of tuberculosis in any other organ. All cases presented with swelling and pain localized to the first MTP joint. A discharging sinus was observed in two cases. The duration of symptoms ranged from six to nine months. All cases exhibited severe joint destruction. In all cases, the diagnosis was established based on histopathological findings of granulomatous inflammation with caseous necrosis obtained from surgical biopsy specimens, and the presence of *Mycobacterium tuberculosis* was additionally confirmed by PCR or culture testing. Standard anti-tuberculous chemotherapy was administered in all cases, and only one case underwent wound debridement. All cases were followed up for only a short duration.

**Table 2:** Summary of reported cases of tuberculosis of the first MTP joint EMB: ethambutol, IC: immunocompromised, INH: isoniazid, MTP: metatarsophalangeal, PCR: polymerase chain reaction, PZA: pyrazinamide, RFP: rifampicin, STM: streptomycin, TB: tuberculosis

Author (year)	Country	Age sex	IC	Other TB involvement	Symptoms at the first MTP joint	Duration of symptoms	Radiographic findings of the first MTP joint	Diagnosis method	Treatment	Outcome	Follow-up period
Davis et al. (1979) [5]	Philippines	80 M	No	None	Swelling and pain, no sinus	9 months	Severe destruction	Surgical biopsy; histology, culture	No description	No description	No description
Sbai et al. (2016) [7]	Tunisia	48 F	No	None	Swelling and pain, no sinus	6 months	Severe destruction	Surgical biopsy; histology	Anti-TB chemotherapy (INH+RFP+PZA+STM × 2 months → INH+RFP × 10 months)	Improved	15 months
Jeong et al. (2016) [6]	Korea	43 M	No	Right lung	Swelling and pain, discharging sinus	6 months	Severe destruction	Surgical biopsy; histology, PCR	Anti-TB chemotherapy (INH+RFP+PZA+EMB × 2 months → INH+RFP+EMB × 7 months)	Ongoing	2 months
Yadav et al. (2023) [8]	India	8 F	No	None	Swelling and pain, discharging sinus	6 months	Severe destruction	Surgical biopsy; histology, PCR, culture	Wound debridement + anti-TB chemotherapy (INH+RFP+PZA+EMB × 2 months)	Lost to follow-up	2 months
Present case	Philippines	31 F	No	None	Swelling and pain, discharging sinus	3 years	Severe destruction	Surgical biopsy; histology, PCR, culture	Metatarsal head resection + anti-TB chemotherapy (INH+RFP+PZA+EMB × 2 months → INH+RFP × 4 months)	Improved	3 years

A notable feature of our case was the use of surgical resection of the first metatarsal head in addition to anti-tuberculous chemotherapy. In skeletal tuberculosis, surgical intervention is typically reserved for cases in which a biopsy is required for definitive diagnosis or when there is extensive osseous involvement or poor response to medical therapy [9]. In this case, surgical intervention was performed both to confirm a definitive diagnosis through biopsy and to treat advanced-stage disease characterized by prolonged symptoms, severe joint destruction, and a discharging sinus. Intraoperatively, the first metatarsal head was found to be extensively replaced by infectious granulation tissue, necessitating its resection for effective infection control. To our knowledge, this is the first report describing resection of the metatarsal head for tuberculosis of the first MTP joint. In previous reports, even in the presence of severe joint destruction, the first metatarsal head was preserved, but all cases lacked mid-term outcome data, leaving uncertainty regarding disease recurrence [5-8]. In our case, combined surgical resection of the first metatarsal head and anti-tuberculous chemotherapy achieved remission over a three-year follow-up period. Resection of the metatarsal head was previously a common procedure for forefoot deformities in rheumatoid arthritis, although postoperative recurrent deformity and impaired toe function were significant concerns [10,11]. In the present case, resection of the first metatarsal head resulted in shortening of the hallux and limited active flexion. However, the patient remains pain-free and able to carry out daily activities without difficulty at three years postoperatively.

Regarding anti-tuberculous chemotherapy, the guidelines jointly issued in 2003 by the Centers for Disease Control and Prevention, the Infectious Diseases Society of America, and the American Thoracic Society recommend a six- to nine-month regimen for all forms of extrapulmonary tuberculosis. This consists of an initial two-month intensive phase using isoniazid, rifampin, pyrazinamide, and ethambutol, followed by a continuation phase of four to seven months with isoniazid and rifampin alone [12]. In the present case, treatment was administered in accordance with this guideline.

## Conclusions

We experienced a rare case of isolated tuberculosis of the first MTP joint in an otherwise healthy young woman without pulmonary involvement. This case underscores the importance of considering skeletal tuberculosis in chronic monoarthritis of the first MTP joint, even in the absence of pulmonary symptoms. For advanced tuberculosis of the first MTP joint, we achieved effective infection control and preserved gait function with only mild impairment during a three-year follow-up period through resection of the metatarsal head combined with anti-tuberculous chemotherapy. This report provides novel insights into the limited literature on foot tuberculosis and highlights the potential role of surgical intervention in cases with severe joint destruction.

## References

[1] Bagcchi S (2023). WHO's global tuberculosis report 2022. Lancet Microbe.

[2] Pigrau-Serrallach C, Rodríguez-Pardo D (2013). Bone and joint tuberculosis. Eur Spine J.

[3] Dhillon MS, Sharma S, Gill SS, Nagi ON (1993). Tuberculosis of bones and joints of the foot: an analysis of 22 cases. Foot Ankle.

[4] Korim M, Patel R, Allen P, Mangwani J (2014). Foot and ankle tuberculosis: case series and literature review. Foot (Edinb).

[5] Davis JA, Bluestone R (1979). Case report 84. Skeletal Radiol.

[6] Jeong JS, Kim JH, Park SH, Jeong JH, Ryu YJ, Moon KW (2016). Tuberculous osteomyelitis of the first metatarsophalangeal joint misdiagnosed as gouty arthritis. J Rheum Dis.

[7] Sbai MA, Benzarti S, Gharbi W, Khoffi W, Maalla R, Khorbi A (2016). An exceptional location of tuberculous arthritis: the metatarsal phalangeal joint. Int J Mycobacteriol.

[8] Yadav S, Rawal G, Jeyaraman M (2023). Isolated tuberculosis of the first metatarsal of the left foot with congenital hallux valgus without pulmonary involvement: a first of its type case. Cureus.

[9] Dhillon MS, Agashe V, Patil SD (2017). Role of surgery in management of osteo-articular tuberculosis of the foot and ankle. Open Orthop J.

[10] Kasai T, Momoyama G, Nagase Y, Yasui T, Tanaka S, Matsumoto T (2021). Disease activity affects the recurrent deformities of the lesser toes after resection arthroplasty for rheumatoid forefoot deformity. Mod Rheumatol.

[11] Matsumoto T, Kadono Y, Nishino J, Nakamura K, Tanaka S, Yasui T (2014). Midterm results of resection arthroplasty for forefoot deformities in patients with rheumatoid arthritis and the risk factors associated with patient dissatisfaction. J Foot Ankle Surg.

[12] (2003). Treatment of tuberculosis. MMWR Recomm Rep.

